# Telomere length in peripheral leukocytes is a sensitive marker for assessing genetic damage among workers exposed to isopropanol, lead and noise: the case of an electronics manufacturer

**DOI:** 10.1186/s41021-021-00226-x

**Published:** 2021-12-16

**Authors:** Yao Lu, Xinxia Liu, Zhiqiang Zhao, Xiaoyan Ou, Yarui Yang, Qing Wei, Jingli Chen, Jun Jiang, Yi Sun, Heping Zhao, Sai Wu, Yun He

**Affiliations:** 1 Department of Toxicology, School of Public Health, Sun Yat-sen University, 74 Zhongshan 2nd Road, Yuexiu District, Guangdong 510080 Guangzhou, China; 2 Academic Department, Southern Medical University, Guangdong Guangzhou, China; 3 Zhongshan Third People’s Hospital, Guangdong Zhongshan, China

**Keywords:** Electronic enterprise, Isopropanol, Lead, Noise, Relative telomere length

## Abstract

**Background:**

Workers in electronics manufacturers may be exposed to various occupational hazards such as isopropanol, lead, and noise. Telomeres are special segments of cap-like DNA protein complex at end of liner chromosomes in eukaryotic cells. Telomere length is a potential marker of genetic damage. The aim of this study is to evaluate the effect of occupational hazards on the relative telomere length (rTL) of peripheral blood cells of workers in an electronics manufacturer, and to explore whether relative telomere length could be a biomarker for assessing genetic damage in the electronics manufacturing industry.

**Methods:**

We investigated a large-scale electronics manufacturer in the Pearl River Delta Region. We ultimately collected 699 qualified workers (248 with isopropanol exposure, 182 with lead exposure, 157 with noise exposure, and 112 controls). During physical examination of the workers, we gave them questionnaires to understand their health statuses and living habits. We also collected peripheral blood samples from these workers to test exposure levels and rTL in the leucocytes.

**Results:**

The concentrations of air isopropanol in all monitored workshops was 25.3 mg/m^3^ and air lead smoke was 0.020 mg/m^3^. The maximum equivalent continuous A sound level noise exposure position was 82.2dB (A). All were lower than those in the Occupational Exposure Limits in Workplaces in China. Urinary acetone in the isopropanol exposed group was 1.04 (0, 1.50) mg/L, and cumulative urinary acetone was 1.48 (0, 5.09) mg-years/L. Blood lead levels (BLLs) were 28.57 (22.77, 37.06) µg/dL, and cumulative blood lead levels (CBLLs) were 92.75 (55.47, 165.13) µg-years/dL. rTL was different between occupational exposed workers and controls: rTL was 0.140 units (95 % CI: 0.022, 0.259) shorter in lead exposed workers and 0.467 units (95 % CI: 0.276–0.658) shorter in noise exposed workers compared to the controls. There is no statistical difference in rTL between isopropanol exposure workers and the controls. In order to elucidate the relationship between rTL and occupational hazards exposure, we divided the isopropanol exposure workers into three groups (0, ~1.43 mg/L, and >1.43 mg/L). None of the rTL difference was statistically significant among exposed workers at different uroacetone levels (*P*>0.05).

The groups with ≥100 µg/dL blood lead had shorter rTL than the group with blood lead below 100 µg/dL (*F*=4.422, *P*=0.013). We incorporated age, gender, birthplace, race, education level, smoking, and alcohol consumption into the linear regression equation. Only blood lead concentration (X) was entered into the regression equation, yielding a multivariate linear regression equation of Y=0.397-0.124X (*F*=8.091, *P*=0.005). Workers with different hearing loss also had statistically significant differences in rTL (*F*=5.731, *P*=0.004). rTL was a protective factor for the occurrence of noise-induced hearing loss (NIHL). The longer the rTL, the lower the risk of NIHL [OR=0.64 (0.42, 0.98)].

**Conclusions:**

rTL was shorter in lead exposed workers and noise exposed workers, and it was a protective factor for the occurrence of the noise-induced hearing loss. Thus, rTL of peripheral blood may be a sensitive marker of genetic damage among workers in environments with lead and noise exposure.

## Introduction

China is a major global manufacturer of electronic products, and the Pearl River Delta is one of the most important clusters of China’s electronics manufacturing industry [1]. During chip cleaning, etching, welding and plating packaging, workers may be exposed to various occupational hazards [2].

Isopropanol is an organic solvent with rare reports of acute or chronic occupational poisoning. It is used as a substitute for methanol in the manufacturing of electronic products. Severe isopropanol poisoning results in CNS and respiratory depression, and circulatory collapse. Recent studies have shown that high-level isopropanol exposure can cause health hazards, such as liver toxicity [3, 4], teratogenicity [5], and reproductive and developmental toxicity [6–8]. But low-level isopropanol exposure-caused potential health hazards are often ignored. Our research team found that even low-level isopropanol exposure could cause 5-hydroxy indole acetic acid (5-HIAA) decreasing, correlated with increased blood pressure [9]. Although the exact mechanism of action of isopropanol has not been fully elucidated, recent studies have suggested that isopropanol affects liver metabolic enzyme activity [10] and promotes the genotoxicity of other substances [11]. The direct evidence of isopropanol’s genetic toxicity is insufficient.

Lead is a ubiquitous and dangerous occupational risk. It is used primarily in welding and plating packaging, and radio element lead spraying operations in the electronics manufacturing industry. Lead can lead to multiple adverse bodily consequences, such as neurotoxicity, cardiovascular toxicity, renal toxicity [12], reproductive toxicity [13], potential carcinogenicity [14] and potentially genetic toxicity [13, 14]. Previous studies have focused more on the health risks caused by high-dose lead exposure, but recently researches have shown that lead is a poison without a threshold, and that even low-level exposure can cause health damage. Okunola et al. reported low-level Pb exposure (0.28-18.94 µg/dL) induced micronucleus in the buccal exfoliated cells of teenagers [15].

Another class of occupational exposure in the electronics manufacturing industry is noise operations. The auditory system, as the main target organ of noise exposure, can manifest as tinnitus, hearing threshold shift or high-frequency hearing loss. It may also appear as irreversible hearing damage and deafness. Noise can also affect the genetic stability. Studies have shown that low-frequency noise diminishes male sperm quality and aggravates DNA damage [16]. Additionally, it has been shown that married women with contact noise of 85~102 dB have elevated chromosome structure distortion rates and monomer fracture [17].

Genetic damage is considered the primary or contributing factor behind many chronic diseases, and can also serve as an indicator of multiple forms of occupational and environmental exposure [18]. Telomere length is a potential marker of genetic damage. Telomere is a DNA-protein complex presenting at the end of eukaryotic linear chromosomes. Studies have shown that telomere length is a factor behind aging [19] and tumorgenesis [20, 21]. Bassig has reported [22] a change in peripheral blood telomere length among individuals exposed to occupational benzene. Additionally, persistent exposure to PM 2.5 has been shown to shorten welders’ peripheral telomere length [23]. Hou [24] found that exposure to high concentrations of air pollution changed peripheral telomere length. Moreover, exposure to ionized radiation has been shown to shorten telomere length in peripheral blood leucocytes [25]. These studies shown that exogenous chemicals can cause changes in telomere length, resulting in genetic damage, which leading to aging and the occurrence of diseases.

Although some studies have focused on workers’ peripheral blood’s telomere length, electronics manufacturing industry workers’ telomere length has yet to receive attention in the scientific literature. Therefore, our study explored the feasibility of peripheral blood leucocytes’ telomere length as a biomarker to reflect genetic damage to workers in the electronics manufacturing industry.

## Methods

### Enrollment and sampling

699 subjects were included in this study, from 2013 to 2014. We classified 587 workers from an electronics factory. This factory mainly produces Information and Communication Technology (ICT) products, including mobile and handheld devices, servers and storage, information equipment, LCD TVs and LCD monitors, network services and communications products. According to their operational sites and workshop monitoring results, we grouped these workers into three groups. The isopropanol exposure workers mainly engaged in maintaining and cleaning products. The lead exposure workers mainly worked in the welding circuit board operations. The noise exposure workers were exposed to steady-state noise such as high-speed patch machine operations, tin paste printing machine operations, test station operations and air compressor operations. The sample included 248 isopropanol exposure workers, 182 lead exposure workers and 157 noise exposure workers. In addition, we selected 112 workers who had participated in the factory’s pre-job physical examination to be the control group.

We designed a structured employee health questionnaire for all subjects. It included basic demographic information, smoking and drinking history, previous diseases, and occupational exposure history. A current smoker was defined as anyone smoking more than one cigarette per day, for at least six consecutive months. A current drinker was defined anyone who had consumed more than one drink per week, for at least six consecutive months. All investigations were conducted by trained investigators.

We collected 5 mL heparin anticoagulant blood and transported it to the laboratory at low temperature. We used 2 mL of blood for blood lead testing. The remaining blood was for extracting genomic DNA. We also collected 50 ml of urine per worker, and stored it at -20 ℃ in a refrigerator for acetone detection.

### The exposure assessment

The Center for Disease Control and Prevention where the factory was located tested external occupational hazard exposure level in the workplace. Isopropanol, lead smoke, acetone, benzene, xylene, and toluene in the air were monitored according to the China National Standard “Determination of Toxic Substance in Workplace Air” (GBZ/T160.48–2007).

We used the Inductively Coupled Plasma detection of Mass Spectrometry (ICP-MS) to measure the subjects’ blood lead concentrations (mg/dL). We took 0.5 ml blood samples after shaking for 10 min to ensure uniformity. Then we added 0.5 ml of HNO_3_ to each blood sample, and heated it in a 100 ℃ water bath for 2~3 h. To determine the final blood lead concentration, we corrected the values by internal calibration.

In order to assess internal isopropanol exposure levels, we measured acetone in the urine according to the China Industrial Standard “Headspace-gas Chromatography Examination Methods for Ethanol, Methanol, N-propanol, Aldehyde, Acetone, Isopropanol and N-butanol in Blood and Urine” (GA/T1073–2013).

### Pure sound hearing test and ear examination

The pure sound hearing test for noise exposure workers referred to the Chinese National Standard “Diagnosis of Occupational Noise-induced Deafness” (GBZ49-2014). The test used an audiometer produced by Interacoustics AS, Denmark, and we calibrated it before formal testing. In an acoustic room with background noise less than 25d B (A), properly trained doctors from the Centers for Disease Control and Prevention tested a total of six frequencies: 500, 1000, 2000, 3000, 4000, and 6000 Hz. Workers had been detached from the noise environment for at least 12 h before inspection. We modified the acoustic listening threshold based on the statistical distributions of age and gender.

An otolaryngologist performed ear examinations on the workers. The examination included bilateral auricle deformity, external ear canal deformity, closure, stenosis, tympanic perforation assessment, subsidence, calcification, overflow, adhesion, etc. We eliminated any workers suffering from the above diseases.

### DNA extraction

We added 3.5 mL of 1×red blood cell pyrolysis to the 2 mL of heparin anticoagulation. After cracking the red blood cell, we centrifuged it in 2000 r/min for 2 min, and then discarded the supernatant. We repeated the steps until no significant red or white blood cell mass was obtained. Then, we added 500µL of 1 ×solution and 20µL of 10 % SDS solution to the cluster, and incubated it at 37℃ for 1 h. We added the protease K 5µL, and stored it at 55℃ overnight. Next, we added 700µL of isopropyl alcohol to the solution. After white flocculation precipitation occurred, we centrifuged it at 12,000 r/min for 8 min, and discarded the supernatant. Then, we added 1.5 mL of 70 % ethanol for cleaning, twice. After instantaneous centrifugation, we discarded the supernatant, and dried it at room temperature for 5 min. Afterword, we added 100µL of 1×Tris-EDTA-Na_2_ buffer at 65℃ for 1 h in order to dissolve the DNA, and then stored it at -80℃.

### Measuring relative telomere length

We measured rTL using quantitative real-time PCR, and expressed it as a ratio of telomere repeat copy number to single copy gene (36B4) copy number. We performed real-time quantitative PCR on the ViiA7 PCR instrument. The telomere primers were: telo upstream: 5’-CGGTTTGTTTGGGTTTGGGTTTGGGTTTGGGTTTGGGTT-3’; telo downstream: 5’-GGCTTGCCTTACCCTTACCCTTACCCTTACCCTTACCCT-3’; for 36B4, they were: 36B4 upstream: 5’-CAGCAAGTGGGAAGGTGTAATCC-3’; 36B4 downstream: 5’-CCCATTCTATCATCAACGGGTACAA-3’. The reaction system (10 µl) contained the following ingredients: 20 ng/µl DNA 0.5 µl, ddH_2_O 3.3 µl, 10µM Upstream primer 0.6 µl, 10µM downstream primer 0.6 µl, SYBRGREEN 5 µl. We initiated the thermal cycling processes at 95 °C for 5 s; and 40 cycles of 1 min at 60 °C, 15 s at 95 °C, and 1 min at 60 °C. Then we randomly selected 100 DNA samples from our participants as standard DNA samples, and constructed a new standard curve in each run to ensure that the amplification efficiency was between 90 % and 110 %. We assayed all samples in triplicate, and calculated the average Cts. The formula for computing rTL was:

$$ {\displaystyle \begin{array}{l}\mathrm{T}/\mathrm{S}={\left[{2}^{\mathrm{Ct}\left(\mathrm{telo}\right)}/{2}^{\mathrm{Ct}\left(36\mathrm{B}4\right)}\right]}^{\operatorname{}1}={2}^{\operatorname{}\Delta \mathrm{CT}};\\ {}\mathrm{rTL}={}^{\operatorname{}\left(\Delta \mathrm{CT}\operatorname{}\Delta \mathrm{CT}\mathrm{standard}\right)}={2}^{\operatorname{}\Delta \Delta \mathrm{CT}}.\end{array}} $$

### Statistical analysis

We conducted all statistical analysis with SPSS 22.0 (IBM SPSS Statistics for Windows, NY, USA). All *P* values were two-sided, and we considered *P* < 0.05 statistically significant. The values were expressed in the form of mean±SD (standard deviation) for the continuous indexes and as percentages with number of cases for the categorical indexes. We then compared the differences between the groups’ basic characteristics with ANOVA or a chi square test.

We used general linear regression to compare blood rTL across three occupational groups (isopropanol exposure workers/lead exposure workers/noise exposure workers vs. controls) by adjusting for potential confounders (described below). Then, we converted rTL and blood lead concentration into a natural logarithm to approach symmetric distribution and included it in the model calculations. We included age (as a continuous variable) in all of the regression models. Additionally, we chose other potential confounders [BMI (continuous variable), smoking and drinking status (categorical variable), education status (as a categorical variable), race and gender (as categorical variables)] based on published studies and general knowledge, and tested them one-by-one in the models. Only the confounders which changed the exposure indexes’ β-estimates by at least 10 % remained.

## Results

### Study subjects’ demographic and clinical characteristics

The study participants’ basic characteristics are presented in Table 1. We ultimately collected 699 qualified samples (248 isopropanol exposed, 182 lead exposed, 157 noise exposed and 112 controls). Gender and race were similar across the four occupational groups. There were no statistically significant differences in job duration or exposure duration (years) between the three occupational exposure groups. The majority of the participants were young (between 18 and 42 years old), however there were slight differences between the groups. The noise-exposed workers were older than the other three groups (*P* < 0.001) and the lead exposed workers were older than the isopropanol-exposed workers (*P* < 0.001) and controls (*P* = 0.04). Age was also similar between the isopropanol-exposed workers and the controls (*P* = 0.218). The differences in body mass index (BMI) between the exposed groups and controls were statistically significant (*P* <0.001), and noise-exposed workers had higher BMI. Smoking and drinking status were also different: there were more current smokers among the noise workers (43.3 %) than among the isopropanol-exposed workers (30.2 %), lead-exposed workers (29.1 %) and controls (30.4 %); and more current drinkers among the noise workers (36.3 %) than among the isopropanol-exposed workers (17.7 %), lead-exposed workers (24.2 %) and controls (8.9 %); neither smoking nor drinking were correlated with rTL (data not shown). Education levels varied between groups, and the lead exposure workers were the most highly educated. rTL was different across the groups (*P*<0.001).

**Table 1:** Demographic and clinical characteristics

Variables	Isopropanol (*n *= 248)	Lead (*n *= 182)	Noise (*n *= 157)	Control (*n *= 112)	*P* value
Male, *n* (%)	207(83.5)	150(82.4)	118(75.2)	83(74.1)	0.066
Age, years, mean±*SD*	24.86±3.85	27.26±6.25	31.98±7.94	25.73±7.44	<0.001
BMI, kg/m^2^, mean±*SD*	21.25±2.93	21.17±3.09	22.33±2.98	20.50±2.87	<0.001
Race Han, *n* (%)	229(92.3)	165(90.7)	149(94.9)	99(88.4)	0.244
Education, *n* (%)					
Middle school or lower	50(20.2)	38(20.9)	13(8.3)	38(33.9)	<0.001
High school	130(52.4)	66(36.3)	129(82.2)	65(58.0)	
College or higher	68(27.4)	78(42.9)	15(9.6)	9(8.0)	
Current smoker, n (%)	75(30.2)	53(29.1)	68(43.3)	34(30.4)	0.018
Current drinker, n (%)	44(17.7)	44(24.2)	57(36.3)	10(8.9)	<0.001
Job duration, years, *M (P25, P75)*	3.0(2.0,4.59)	2.8(1.7,4.0)	3.0(1.7,5.0)	-	0.391
Exposure duration, years, *M (P25, P75)*	3.0(1.84,4.6)	3.0(1.92,4.9)	3.0(1.7,5.0)	-	0.867
rTL, *M (P25, P75)*	1.06(0.71,1.43)	0.88(0.64,1.48)	0.69(0.38,1.01)	1.07(0.84,1.28)	<0.001

*BMI* body mass index, *rTL* relative telomere length

### Exposure levels in the occupational groups

Air monitoring results of occupational hazard factors are presented in Table 2. The concentrations of air isopropyl alcohol in all monitored workshops and time points were 25.3 mg/m^3^. Thus, we can regard this contact level as low-level exposure, compared to the occupational exposure limit (700 mg/m^3^). The lead smoke air test results were 0.020 mg/m^3^, lower than the Occupational Exposure Limit (0.03 mg/m^3^) for Hazardous Factors in Workplaces. The cumulative exposure level, that is the product of job durations versus exposure concentration, were used to represent the cumulative level of exposure. Urinary acetone in the isopropanol exposed group was 1.04 (0, 1.50) mg/L, and cumulative urinary acetone was 1.48 (0, 5.09) mg-years/L. Blood lead levels (BLLs) were 28.57 (22.77, 37.06) µg/dL, and cumulative blood lead levels (CBLLs) were 92.75 (55.47, 165.13) µg-years/dL. Other organic components in the workplace, including benzene, acetone, xylene, toluene and methanol, were not detectable.

**Table 2:** Air monitoring results of occupational hazard factors

Hazard factors	Sampling Type	Allowable concentration (mg/m^3^)	Minimum detected concentration (mg/m^3^)	Results(mg/m^3^)	
				Minimum	Maximum
isopropanol	Short-time contact concentration	700	0.7	25.0	50.7
lead	Time-weighted average concentration	0.03	0.003	0.016	0.023
Acryketone	Short-time contact concentration	450	1	Undetected	
Benzene	Short-time contact concentration	10	0.4	Undetected	
DiMethylene	Short-time contact concentration	100	0.3	Undetected	
Methene	Short-time contact concentration	100	0.3	Undetected	

Environmental noise intensity in noise positions are presented in Table 3. According to Ministry of Health standards, <85 dB of working environment noise (A) is judged as qualified site, and ≥ 85 dB (A) is judged an unqualified site. The monitoring results showed that neither site exceeded the national standards.

**Table 3:** Environmental noise intensity in noise position

	Intensity^a^	
Positions	Minimum	Maximum
Plug-in Machine Operation	73.8	80.8
High-speed patch er operation	72.3	76.0
Air compressor operation	81.2	82.2
Tin paste printing press operation	71.0	76.1
Test Station Operation	76.3	82.1

^a^an equivalent continuous A sound level, with a national standard of 85dB (A)

### Differences in relative telomere length in four occupational groups

**Fig. 1:**
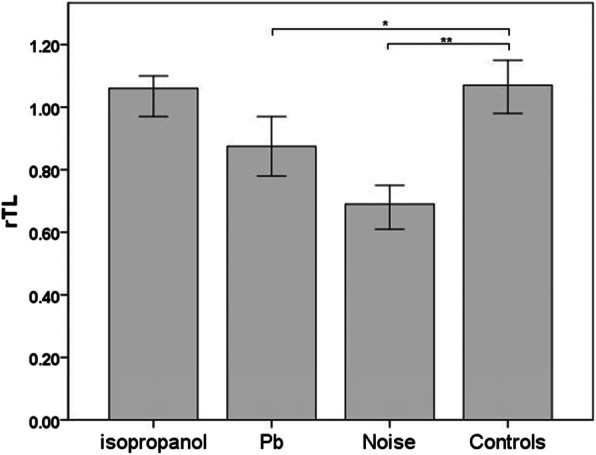
Telomere length changed among different occupational groups. Longitudinal bar indicated median values. Each group compared to controls with adjusted linear regression model. * *P*< 0.05; ** *P* < 0.001

A multivariate linear regression model showed that the occupationally exposed workers’ relative telomere length was different from that of the controls (Table 4): rTL was 0.21 units (95 % CI: 0.022, 0.259) shorter in lead-exposed workers and 0.467 units (95 % CI: 0.276–0.658) shorter in noise-exposed workers than in the controls. Additionally, there was no statistical difference between telomere lengths in isopropanol exposure workers and the controls (Table 5). The control workers had the longest telomere length, second was the isopropanol exposure group, followed by the lead exposure group, and the shortest was the noise exposure group (Fig. 1).

**Table 4:** Differences of rTL^a^ in four occupational groups

	Partly adjusted^b^		Fully adjusted^c^	
Occupational groups	β(95 %CI)	*P* value	β(95 %CI)	*P* value
Isopropanol	-0.057(-0.155, 0.041)	0.252	-0.064(-0.166, 0.038)	0.218
Pb	-0.111(-0.224, 0.002)	0.054	-0.140(-0.259, -0.022)	0.013
Noise	-0.497(-0.674, -0.320)	<0.001	-0.467(-0.658, -0.276)	<0.001
Controls	Ref.	-	Ref.	-

^a^rTL log-transformed

^b^Partly adjusted linear regression model: only adjusted for age

^c^Fully adjusted linear regression model: adjusted for age, gender, race, education status, BMI, smoking and drinking status

### Telomere length in isopropanol-exposed workers

In order to elucidate the relationship between telomere length and exposure to occupational hazards, we divided the isopropanol exposure workers into three groups (0, ~1.43 mg/L, and >1.43 mg/L). There was no statistically significant difference between telomere length among exposed workers at different uroacetone levels (*P*>0.05). Additionally, there was no statistically significant relationship between urinary acetone concentration and association with telomere length after controlling for mixed factors such as age, gender ethnicity, education level, smoking and drinking (*r*=-0.027, *P*=0.69).

**Table 5:** Comparison of telomere length in isopropanol exposure workers

Isopropanol exposure workers	*N*	rTL, *M (P25, P75)*	*F*	*P* value
urinary acetone, mg/L				
0	84	1.07(0.71,1.44)	0.894	0.532
~1.43	51	1.10(0.68,1.42)		
>1.43	50	1.10(0.70,1.67)		
cumulative urinary acetone, mg-years/L				
0	84	1.07(0.71,1.44)	1.187	0.306
~4.36	51	1.22(0.84,1.67)		
>4.36	50	1.09(0.64,1.32)		

### Telomere length in lead-exposed workers

**Fig. 2:**
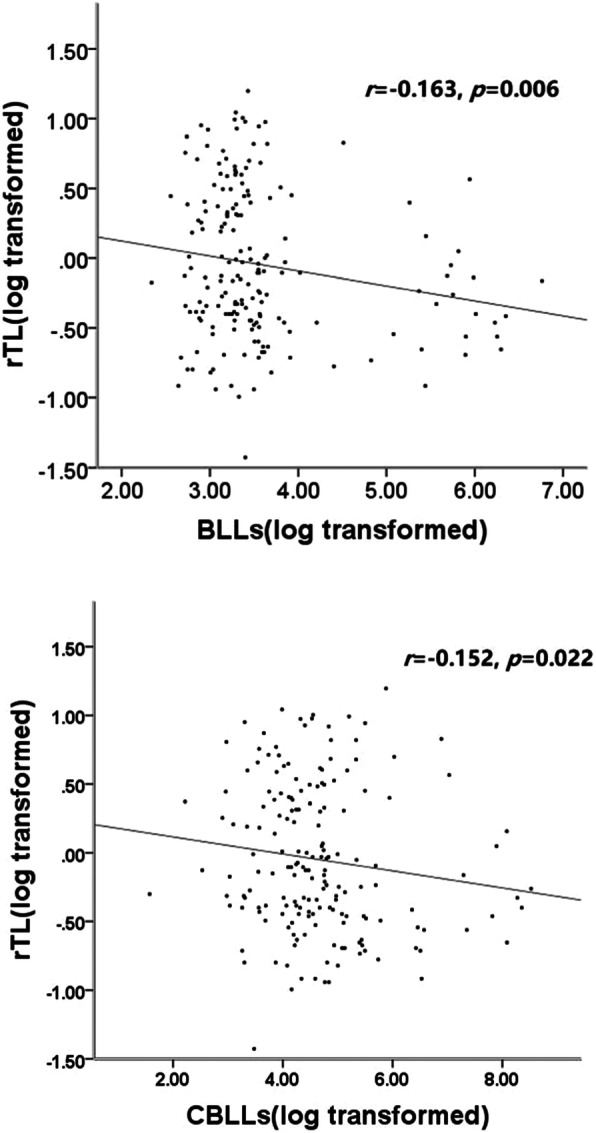
Partial correlation analysis was used to explore the association between BLLs(log transformed), CBLLs(log transformed) and rTL(log transformed) in Pb exposed workers. Age, gender, race, education status, BMI, smoking and drinking status was adjusted

Comparison of telomere length in Pb exposure workers is presented in Table 6; Fig. 2. We also divided the lead-exposed workers into three groups (<25, 25~100, >100 µg/dL) or (<67, 67~323, >323 µg-years/dL). The group with >100 µg/dL had shorter telomere length than the group with <100 µg/dL, (*F*=4.422, *P*=0.013). We incorporated age, gender, birthplace, education level, education, job durations, smoking, and alcohol consumption into the linear regression equation. Only blood lead concentration (X) was entered into the regression equation, yielding a multivariate linear regression equation of Y=0.397 – 0.124X (*F*=8.0917, *P*=0.005). This showed that the relative telomere length had declined by 0.124 units after each unit increase in blood lead concentration.

**Table 6:** Comparison of telomere length in Pb exposure workers

Pb exposed workers	*N*	rTL, *M(P25,P75)*	*F*	*P* value
BLLs, µg/dL				
<25	62	0.95(0.68,1.48)	4.422	0.013
25~100	97	0.90(0.64,1.57)		
>100	23	0.77(0.52,0.88)		
CBLLs, µg-years/dL				
<67	62	1.08(0.68,1.55)	2.447	0.089
67~323	97	0.85(0.63,1.38)		
>323	23	0.67(0.57,1.17)

### Telomere length in noise-exposed workers

Comparison of relative telomere length of workers with different hearing loss levels is presented Table 7. Hearing loss at 3,000 Hz, 4000 Hz, or 6000 Hz is considered high-frequency hearing loss. Hearing loss at 500 Hz,1000 Hz, or 2000 Hz is defined as low-frequency hearing loss. Workers with different forms of hearing loss also had varying relative telomere length. These differences were statistically significant (*F*=5.731, *P*=0.004). Also, workers with speech-frequency hearing loss had the shortest rTL.

**Table 7:** Comparison of relative telomere length of workers with different hearing loss levels

Groups	*N*	rTL	*F*	*P* value
Normal hearing	60	0.76(0.46,1.03)	5.731	0.004
High-frequency hearing loss of double ears or single ear	85	0.68(0.31,1.06)		
Speech-frequency hear loss of double ears or single ear	12	0.49(0.11,0.63)		

Previous research has suggested that telomere length may influence NIHL. Therefore, we used Multi-classification Ordered Logistic Regression analysis to explore the relationship between the workers’ NIHL and their telomere length (Table 8). Parallel line tests showed that the regression equations were parallel (*P*> 0.05). Therefore, we entered age, gender, smoking, drinking and telomere length into the fitting model. Age was a risk factor for the NIHL occurrence [OR=1.08 (1.03, 1.14)]. Gender was also a risk factor for NIHL, and there was a higher risk of NIHL among women [OR = 2.45 (1.09, 5.49)]. However, rTL was a protective factor for NIHL occurrence. The longer the rTL, the lower the risk of NIHL [OR=0.64 (0.42, 0.98)].

**Table 8:** Potential influencing factors for NIHL

Variable^a^	*β*	*β S.E.*	Wald *χ*^*2*^	*P* value	*OR*(95 %*CI*)
Age	0.077	0.0257	8.958	0.003	1.08(1.03,1.14)
rTL	-0.448	0.217	4.264	0.039	0.64(0.42,0.98)
gender	0.896	0.412	4.730	0.030	2.45(1.09,5.49)
Current smoker, n(%)	-0.442	0.363	1.482	0.223	0.643(0.315,1.309)

^a^variable assignment: double ear hearing normal =0, double ear high frequency increase =1, and language frequency increase =2. Self variable assignment: gender: male =0, female =1; smoking: smoking =0, not smoking =1; alcohol: drinking =0, not drinking =1

## Discussion

Telomere is a small segment of the DNA-protein complex present at the end of linear chromosomes in eukaryotic cells. It can maintain chromosomal integrity and control the cell separation cycle. In this study, we investigated the effects of three common occupational risks in electronics manufacturing on the relative telomere length of peripheral blood leukocytes in workers. The results indicated that the control workers had the longest telomere length, followed by the isopropanol exposure group, and then the lead exposure group. However, they had the shortest telomere length in the noise exposure group. In short, worker telomere length influences occupational exposure levels.

Noise in operating environments in the electronics manufacturing industry is primarily steady-state. Its intensity is far lower than that of exposure in logging, mining and heavy machinery, and generally its equivalent continuous A sound level is about 70-80 dB (A). The maximum noise exposure intensity in the present study was 82.2 dB (A), meeting the national standard of >85dB (A). Reports on the relationship between noise and telomere length are scarce. In 2015, Park [26] conducted research in a community of nearly 3,000 people to uncover the relationship between “neighborhood quality” and telomere length. “Neighborhood quality” was evaluated based on three variables: noise, insecurity and destruction of public property. After adjusting for potential confounders such as gender, age, income, lifestyle and social class, they found that people with poor “neighborhood quality” had shorter telomere length, yet they did not separately demonstrate the effect of noise on telomere length. Our study showed that noise exposed workers had shorter telomere length than other occupationally-exposed workers (*P* <0.05). Because we did not determine individual exposure for noise workers, we could not classify workers according to noise exposure intensity. Instead, we grouped workers by the degree of noise-induced hearing loss, and found that exposed workers with hearing loss had shorter telomeres than workers with normal hearing. The telomeres of noise-exposed workers with speech frequency hearing loss were shorter than those of the noise-exposed workers with high-frequency hearing loss. This suggests a correlation between hearing loss effect and telomere length caused by noise exposure. As such, telomere length might be an effective biomarker of noise exposure.

In 2012, Wu [27] first reported that occupational lead exposure reduces the relative telomere length of peripheral blood. Wu found that workers high levels of lead exposure and blood lead concentration of 854.59±558.56 mg/dL had shorter peripheral blood telomere length than normal workers (<400 mg/dL). However, the study did not clarify the effect of low concentration lead exposure on telomere length. Our results showed that workers with lower lead exposure concentrations [28.57 (22.77, 37.06) µg/dL] could experience telomere shortening. These results are consistent with findings in exposed workers with high blood lead concentrations. They suggest that the relative telomere length of peripheral blood leukocytes could be used to identify early and low-concentration lead exposure. Multivariate linear regression results showed that the blood lead concentration (X) had entered the regression equation Y=0.397-0.124X (*F*=8.091, *P*=0.005). There was also a dose-effect relationship between the blood-lead concentration and telomere length. In 2015, Zota [28] analyzed the relationship between blood lead levels relative telomere length based on data from the National Health and Nutrition Survey (1999-2002). Zota found no correlation between a blood lead concentration of 1.67 mg/dL (95 %CI: 1.63,1.70) and telomere length. However, Pawlas [29] found that blood lead concentration [3.28(0.90-14.2) mg/dL] in 99 8-year-old children was negatively correlated with relative telomere length in the peripheral blood. The relationship between lead exposure and the length of the peripheral blood telomere has been supported by further research and evidence.

Isopropanol is widely used in the electronic manufacturing industry as cleaning agents. Severe isopropanol poisoning results in CNS and respiratory depression, and circulatory collapse. However, studies on low-level isopropanol occupational exposure are rare. The previous study of our research group found that even low-level isopropanol exposure increased blood pressure. It suggested that isopropanol, which has long been considered “safe”, has potential hazards. In this study, we found no significant difference in telomere length between isopropanol exposed workers and pre-job workers. Although blood pressure was increased in isopropanol workers at similar exposure concentrations, genetic damage effects were not observed. This might because the exposure concentrations of isopropanol and its metabolite acetone and pyruvate were too low to cause a genetic damage effect. Mitran et al. found that acetone exerted neurotoxic effects on workers exposed to it and acetone exposure slowed down nerve conduction velocity and elevated action protential latency in the peroneal nerve [30]. And another study demonstrated that liver, kidneys and bone marrow were the primary target sites of acetone toxicity [31]. But in essence, toxicity developed only at high doses of acetone. Toxicity studies of low-concentration isopropanol exposure are still needed to support this hypothesis.

Shorter telomere length is correlated with increased incidence of age-related diseases such as diabetes, hypertension and other cardiovascular diseases [32]. For example, endothelial cell dysfunction is an early pathological change in cardiovascular disease. Bhayadia [33] found that endothelial-dependent vasodilation decreased in telomerase-deficient mice, while antioxidants could reverse this endothelial dysfunction. This indicated oxidation stress’s important role in this dysfunction. On the other hand, both lead and noise exposure can also activate oxidative stress by reactive oxygen free radicals, which damage biomacromolecules such as DNA [34]. Thus, oxidative stress may play a role in lead and noise exposure and the shortened relative telomere length of peripheral blood. Zhang [35] reviewed the relationship between several exogenous chemical exposures to telomere length and discussed potential mechanisms for telomere change in exogenous chemistry. Zhang determined that exogenous chemicals had induced oxidative stress and chronic inflammation, further shortening telomeres through gene regulation and epigenetic changes. Telomere shortening can be maintained or corrected by upgrading telomerase activity and activating telomere retention mechanisms. The imbalance between the telomere retention mechanism and the telomere shortening mechanism leads to telomere shortening, which leads to chromosome instability, aging, cell dysfunction and damage, and eventually occupational exposure-related diseases. In our study, the results for lead exposure and noise exposure workers fit the hypothetical model proposed by Zhang. Thus, telomere length might be an early effect biomarker of lead and noise exposure.

## Conclusions

In the electronics manufacturing industry, the control workers had the longest telomeres, followed by the isopropanol exposure group, and then the lead exposure group. The shortest telomeres were those of the noise exposure group. Workers with high levels of lead exposure had shorter peripheral blood relative telomere length than those with low levels of lead exposure. Additionally, we found that relative telomere length is a protective factor for the occurrence of noise-induced hearing loss. As such, relative telomere length in peripheral blood could be a sensitive marker of genetic damage for workers in environments with lead and noise exposure.

## Data Availability

All data generated or analyzed during this study are included in this published article and its supplementary information files.

## Abbreviations

rTL: Relative telomere length
BLLs: Blood lead levels
CBLLs: Cumulative blood lead levels
ICP-MS: Inductively Coupled Plasma detection of Mass Spectrometry
SD: Standard deviation
BMI: Body mass index
